# Transabdominal and transvesical laparoscopic correction of vesico-vaginal fistula: 42 cases experience

**DOI:** 10.1590/S1677-5538.IBJU.2018.0743

**Published:** 2020-01-10

**Authors:** Aurus Dourado Meneses, Adriane Queiroz Oliveira, Daniel Alencar de Araújo, Denise Teixeira Santos, Larisse Yara de Carvalho, Walberto Monteiro Neiva Eulálio, Zhamshid Okhunov, Juan Gómez Rivas, Domenico Veneziano, Mirandolino Batista Mariano

**Affiliations:** 1 Departamento de Urologia Hospital São Marcos TeresinaPI Brasil Departamento de Urologia do Hospital São Marcos, Teresina, PI, Brasil;; 2 Centro Universitário UNINOVAFAPI TeresinaPI Brasil Centro Universitário, UNINOVAFAPI, Teresina, PI, Brasil;; 3 Universidade Federal do Piauí TeresinaPI Brasil Universidade Federal do Piauí - UFPI, Teresina, PI, Brasil;; 4 Department of Urology University of California Irvine EUA Department of Urology, University of California, Irvine, EUA;; 5 Department of Urology Hospital Universitario La Paz Madrid Espanha Department of Urology, Hospital Universitario La Paz, Madrid, Espanha;; 6 Department of Urology Reggio Calabria Italy Department of Urology, Reggio Calabria, Italy;; 7 Departamento de Urologia Hospital Santa Casa de Misericórdia Porto AlegreRS Brasil Departamento de Urologia, Hospital Santa Casa de Misericórdia, Porto Alegre, RS, Brasil

## Abstract

**Introduction and Objective:**

Several methods and techniques have been described for the treatment of vesicovaginal fistula (VVF) including abdominal, vaginal and endoscopic approaches. The development of laparoscopic surgery minimizes the morbidity associated with laparotomy, reducing the period of convalescence, being increasingly used in the management of VVF. This aim of this study is to present 42 cases of laparoscopic vesicovaginal fistula repair and to evaluate their results.

**Materials and Methods:**

Forty-two patients with a diagnosis of VVF between 1998 and 2016 were included, with precise indications of abdominal surgical approach as recommended by Lee et al. (1) Cystoscopy, Retrograde urethrocystography and excretory urography confirmed the presence of VVF and ruled out ureteral lesions in all patients.

**Results:**

Forty-two patients with VVF, mean age of 40.35 years (19-75 years), were treated. The most frequent cause of VVF was abdominal hysterectomy (80.95%) 34 patients (80.95%) had never been treated, while 7 patients (16.66%) had undergone unsuccessful abdominal surgical treatment. One patient (2.38%) underwent three attempts of correction, one vaginally and two abdominal without success. The average time of hospitalization was 3 days. The average duration of the vesical catheter was 12 days. Complications occurred in 4 patients (9.52%). Only 2 patients (4.76%) had recurrence at 40 and 90 days after their first surgery, both of them were previously submitted to radiotherapy.

**Conclusion:**

The laparoscopic approach of VVF is an excellent alternative to the traditional abdominal approach. Therefore, it is a feasible, effective and minimally invasive method that can treat this entity.

Available at: http://www.intbrazjurol.com.br/video-section/2018743_Meneses_et_al
